# Splints for Hip Disease

**Published:** 1861-06

**Authors:** 


*Splints for Hip Disease.*—Very excellent splints to keep up extension in hip disease have been devised by Drs. Davis and Sayre, of New York. These are used in all stages of the disease. In the early months of the attack, the extension takes away the pressure of the inflamed head of the femur in the joint, and allows it to recover. After an operation, it is used to prevent too great shortening while the end of the femur is contracting a ligamentous adhesion to the side of the pelvis.

Sayre's splint is applied to the outer side of the limb, and supports the body by a perineal band, and is attached to the side of the leg, just above the ankle, by spiral adhesive straps.

There are, however, some imperfections about this excellent instrument.

The principal of these is the fact, that it is applied to the outer side of the limb, and hence, comes directly over the ulcer, which remains for a time after the operation. The ulcer is apt to be injuriously pressed upon, and the padding of the splint becomes fouled with the discharges.

Before Sayre's splint came to my notice, I had already devised one, which, after considerable experience, I am disposed, decidedly, to prefer: It is applied to the inner, instead of the outer side of the limb, and is therefore, quite out of the way of the wound of the operation. The top supports the perineum by a crutch piece, padded and covered with patent leather, and the bottom is attached firmly to the sole of the shoe, and at each step transmits the weight of the body directly to the ground. The extension is made by a screw working in a tube. If the limb requires to be kept well down, it is done with the utmost comfort by means of broad adhesive straps applied to each side of the leg, as in cases of strap extension in fracture, and tied under the shoe, or buckled to its side.

The price of the whole is five dollars; (that of the Sayre's splint, fifteen dollars.) They can be made in this city to order, by simply sending a measure of the length of the patient's foot, of the distance from the perineum to the sole of the sound foot, and a statement of whether the disease is on the right or left side.

